# Stability and Dynamics of Milling Process During Cutter–Workpiece Engagement and Disengagement Stages

**DOI:** 10.3390/mi17060738

**Published:** 2026-06-18

**Authors:** Jiawei Mei, Chengzhu Wu, Ye Jin, Luxuan Sun, Sunyi Liu, Yaoxuan Han, Yuyang Huang

**Affiliations:** 1 School of Mechanical, Electronic and Control Engineering, Beijing Jiaotong University, Beijing 100044, China; jwmei@bjtu.edu.cn (J.M.); 25126133@bjtu.edu.cn (Y.H.); 2 School of Aerospace, Faculty of Science & Engineering, University of Nottingham Ningbo China, Ningbo 315100, China; chengzhuwu.carlos@gmail.com (C.W.); jinwendy09@gmail.com (Y.J.); ssyls2@nottingham.edu.cn (L.S.); 3 Faculty of Computer and Mathematical Sciences, The Hong Kong Polytechnic University, Hong Kong 999077, China; nosiris117@gmail.com

**Keywords:** milling dynamics, tool engagement, tool disengagement, cutting forces

## Abstract

In milling operations, cutters entering and exiting workpiece boundaries cause varying radial immersions and chip thicknesses. This generates aperiodic cutting forces that often induce vibrations and degrade surface quality. To address this, this study aims to accurately predict milling forces and surface profiles during these critical engagement and disengagement phases. An analytical approach was developed to estimate the changing distances between the cutting teeth and workpiece boundaries, enabling the precise calculation of the dynamic chip thickness as the cutter transitions through the material. Based on these dynamic calculations, milling forces and system responses were simulated. Experimental validation demonstrated a strong agreement between the simulated cutting forces, machined surface profiles, and real-world results. Notably, findings revealed that even cutting parameters deemed stable by traditional stability lobes can still trigger vibrations during these boundary transitions. Consequently, a novel parameter selection strategy is proposed to effectively prevent these transient vibrations, significantly enhancing the final surface finish. Ultimately, this comprehensive modelling framework provides a deeper understanding of the system dynamics throughout the entire milling process, offering high relevance for broader applications, such as optimising energy consumption, predicting tool wear, and improving machining parameter optimisation.

## 1. Introduction

Milling is a two-century-old and important machining or cutting process that employs a milling cutter, a rotary cutting tool, to remove material from the surface of a workpiece [1,2]. Milling operations have been extensively used to machine sculptured surfaces [3], thin-walled parts [4], and composite materials [5] due to the fact that they can produce high precision and accuracy components in a cost-effective and efficient way, and their machining performance can be further improved to achieve sufficient surface finish and desired dimensions/shapes with the application of ultrasonic vibration assistance [6,7], high-speed rotating spindles, and variable helix and pitch cutters [8,9,10]. Wavy machined surfaces and system vibrations, however, always occur due to the discontinuous cut nature of milling processes, significantly limiting the machined surface integrity and tool service life [11]. Understanding the prediction and avoidance of milling vibrations is therefore necessary [12,13]. Accurate chip thickness estimation, which could be strongly influenced by the radial engagement in the cut, is essential in milling dynamics simulation and vibration analysis [14,15]. Cutting-engaged areas should therefore be calculated in the first stage of milling dynamics simulations [16].

**Figure 1:**
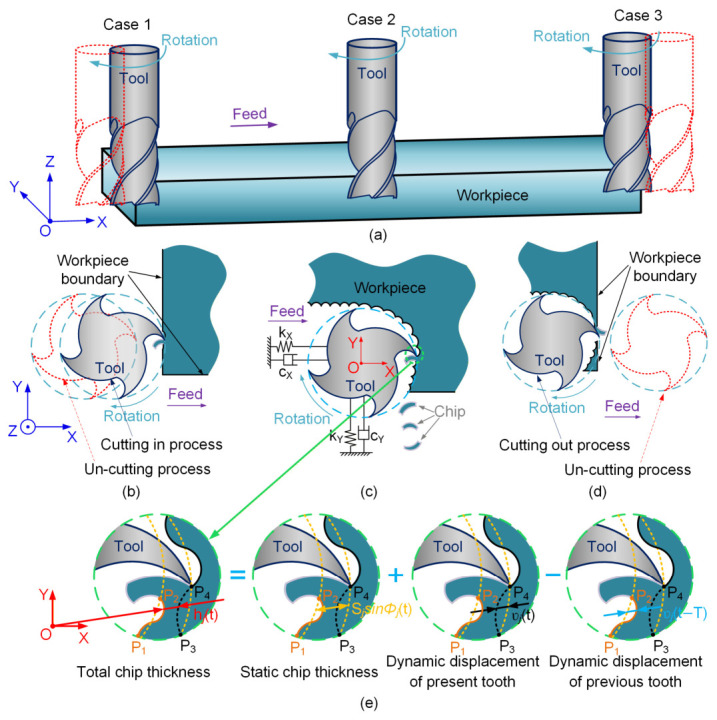
Typical milling dynamics conditions, including: (**a**) a general view of the milling process; cross sections of the engagement and disengagement phases when a cutter (**b**) is starting to contact a workpiece, (**c**) is stably performing cutting, and (**d**) is beginning to disengage/escape the workpiece; and (**e**) a schematic diagram of a self-exited vibration in the milling process.

A typical milling process has three stages (see Figure 1d). We define stage 1 as the initial engagement process (see case 1 in Figure 1a), where a cutter begins to enter a workpiece and the cutting-engaged area increases from zero to a desired radial depth of cut. In this process, after the cutting edge first touches the workpiece boundary and starts cutting into the material (see the cross sections of the cutter and workpiece during this process in Figure 1b), the entry and exit angles of the tool corresponding to each spindle revolution vary frequently due to the relative position of the cutter and workpiece.

We define stage 2 as the normal milling process, where the cutting engagement is always considered to be the desired radial depth of cut (see case 2 in Figure 1a). In this process, the entry and exit angles are constant and can be calculated based on the radial immersion of the cutter.

We define stage 3 as the final disengagement process (see case 3 in Figure 1a), where the cutter exits the workpiece, and the engagement area decreases from the desired radial immersion to zero. In this process, after the cutting edge first cuts out the workpiece boundary and starts exiting the workpiece (see the cross sections of the cutter and workpiece during this process in Figure 1d), the un-cut material in the feed direction becomes less until zero, resulting in the continuous change in the cutting engagement, and hence, the system dynamic reaction may also vary.

Milling mechanics studies have been mainly focused on stage 2 as aforesaid, where the radial depth of cut is constant; hence, the cutting-engaged area can be easily confirmed. For example, the Altintas regenerative chatter model [11], taking into account the self-excited effect, has been utilised and updated by various researchers to estimate the chip thickness and predict system dynamics [17,18,19,20,21] to simulate milling forces that can sufficiently match their experimental results. Based on the regenerative chatter model, stability lobes have been proposed through frequency-domain [22,23] and time-domain [24,25,26] methods to predict stable machining parameter regions and are accepted as one of the most common strategies to avoid chatter vibrations [27]. In these models, the chip thickness, $h_{j}(t)$, removed by the tooth (*j*) at time *t*, is divided into a static part (influenced by the feed of the cutter) and a dynamic part (affected by the regenerative mechanism) and can be derived as:

(1) $$h_{j}\left(t\right)=f_{j}sin\emptyset_{j}\left(t\right)+\upsilon_{j}\left(t\right)-\upsilon_{j}\left(t-T\right),$$

where $f_{j}sin\emptyset_{j}\left(t\right)$ is the static chip thickness (see Figure 1e), and $\upsilon_{j}\left(t\right)$ and $\upsilon_{j}\left(t-T\right)$ are the dynamic displacements (see Figure 1) caused by system vibrations.

The aforementioned models and methods are only effective for normal milling procedures, where the cutting entrance and exit angles are fixed [28]. The cutting engagement, and thus the entry and exit angle, vary dramatically [29] in cutting stages 1 and 3 when the cutter enters and exits the workpiece boundaries, affecting the cutting continuity and chip thickness and, consequently, the milling force and system dynamics response. This severe cutting engagement variation not only limits the milling performance and the machined surface quality but also makes dynamics modelling in milling stages 1 and 3 more challenging and difficult than in stage 2. Only a limited number of studies have been reported concerning the mechanics in milling stages 1 and 3, including:

(i) The investigation of the effect of the in-plane exit angle on burr forming for face milling [29], although the system dynamics was not considered.
(ii) The development of a time-domain cutting force model considering when the cutter exits the workpiece boundaries in a slot milling process, based on the assumption that the cutter first exits the workpiece boundaries in the feed direction [27], although this model only works for the slotting process and cannot be expanded to milling conditions with other radial immersions.
(iii) The proposal of a dynamic milling force and displacement simulation method considering when the cutter enters the workpiece [28], although the prediction accuracy requires further improvements, and there is a lack of studies on machined surface profile estimation and milling cutter vibration suppression when the cutter enters and exits the sides of the workpiece.

In summary, milling forces calculated from most existing models can only predict the dynamic responses in milling stage 2, and machining parameters under the stability lobe calculated for milling stage 2 may not be appropriate for application in milling stages 1 and 3, as these parameters might sometimes lead to unexpected vibrations [27,28]. Therefore, a new milling dynamics model taking into account milling stages 1 and 3 is required to evaluate the relevant dynamic responses and suggest proper cutting parameters. To this end, a new model is proposed to simulate cutting forces in the time domain considering when the milling cutter enters and exits the sides of the workpiece.

The model initialises and then updates the distances from the cutting teeth to the workpiece boundaries at each time step in the simulation, enabling the estimation of the instantaneous cutting-engaged area at each instant, thereby contributing to the prediction of the frequently varied chip thickness. Based on this prediction, the calculations of the system dynamic reactions are derived, and an analytical method is then developed to predict machined surface profiles.

Furthermore, to suppress vibrations in these specific processes, a strategy that can be utilised to plot the cutter’s dynamic displacements relative to the workpiece under different machining conditions is presented to suggest proper milling parameters. Experimental validations indicate that even the parameters that ensure a stable machining condition in a normal cutting operation may cause vibrations during the engagement and disengagement processes when a cutter enters and exits the workpiece boundaries, resulting in vibration marks on machined surfaces.

Satisfactory agreements are observed between the measured and simulated forces, and the suggested parameters obtained from the proposed strategy led to improved milling stability and a step-improved surface integrity.

## 2. Mechanics of Entire Milling Process

### 2.1. Modelling System Dynamics in Normal Milling Process

Normal milling processes are illustrated as case 2 in Figure 1a, where the cutting-engaged area remains constant and depends solely on the desired radial depth of cut [11]. To analyse the dynamics, a cross section of the cutter with two orthogonal degrees of freedom (DoF) is first considered (see Figure 1c). In this 2D machining system, after a cutting tooth contacts the workpiece and causes milling forces, the system dynamics can be represented as two differential equations:

(2) $$\left\{\begin{array}{c} \ddot{x}+2\xi_{x}\omega_{nx}\dot{x}+\omega_{nx}^{2}x=\frac{\omega_{nx}^{2}}{k_{x}}F_{x}\left(t\right)\\ \ddot{y}+2\xi_{y}\omega_{ny}\dot{y}+\omega_{ny}^{2}y=\frac{\omega_{ny}^{2}}{k_{y}}F_{y}\left(t\right) \end{array}\right..$$

Due to the machining forces acting on the cutting area, dynamic displacements between the cutter and the workpiece appear on both the X and Y axes and, hence, leave a wave surface on the workpiece. Therefore, 2D machined surfaces produced by previous and current teeth can be represented as arc $\overset{⏜}{P_{1}P_{2}}$ and arc $\overset{⏜}{P_{3}P_{4}}$ (see Figure 1e), respectively, indicating that the wave surface left by the previous tooth (i.e., arc $\overset{⏜}{P_{1}P_{2}}$) is removed by the present tooth and a new wave surface (i.e., arc $\overset{⏜}{P_{3}P_{4}}$) is produced. Consequently, the chip generated by the current tooth is the material between arc $\overset{⏜}{P_{1}P_{2}}$ and arc $\overset{⏜}{P_{3}P_{4}}$ (see Figure 1e), which forms the basis for calculating the chip thickness, as shown in Equation (1), where the static part caused by the feed of the cutter (i.e., $S_{j}sin\emptyset_{j}\left(t\right)$) and dynamic parts caused by the previous tooth ($\upsilon_{j}\left(t-T\right)$) and present tooth ($\upsilon_{j}\left(t\right)$) are involved. Then, the differential tangential and radial milling forces can be expressed as:

(3) $$\left\{\begin{array}{c} dF_{t,j}(t)=K_{tc}h_{j}\left(t\right)da+K_{te}da\\ {dF}_{r,j}(t)=K_{rc}h_{j}\left(t\right)da+K_{re}da \end{array}\right..$$

To estimate the system dynamics under the forces calculated in Equation (3), $dF_{t,j}(t)$ and ${dF}_{r,j}(t)$ need to be transformed into X and Y axes:

(4) $$\left[\begin{array}{c} {dF}_{x,j}\left(t\right)\\ {dF}_{y,j}\left(t\right) \end{array}\right]=\left[\begin{array}{cc} -cos\emptyset_{j}\left(t\right) & -sin\emptyset_{j}\left(t\right)\\ sin\emptyset_{j}\left(t\right) & -cos\emptyset_{j}\left(t\right) \end{array}\right]\left[\begin{array}{c} dF_{t,j}\left(t\right)\\ {dF}_{r,j}\left(t\right) \end{array}\right].$$

**Figure 2:**
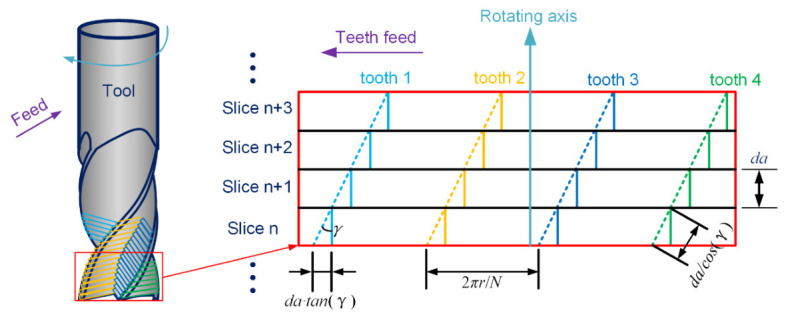
A schematic diagram of a discretised 4-tooth helical cutter and its unrolled view, where the cutter is sectioned into a number of slices.

For a helical milling tool, its helix angle causes the phenomenon that the full length of the cutting edge does not align at the same rotation angle [11]. To model this phase delay and capture its impact on milling forces, the tool is discretised into multiple slices with the same thickness (da) along the axis of the rotation, and the periphery of the tool is ‘unrolled’ onto a 2D surface (see Figure 2). Thus, the helical cutting edges can be transformed into straight lines with a slope (γ) corresponding to the helix angle, and the angular delay between the adjacent slices of the same tooth can also be calculated as da·tan(γ)/r (note that r is the radius of the cutter). By defining the rotation angle corresponding to the bottom of tooth 1 ($\emptyset_{1,1}(t)$), the specific rotation angle corresponding to the slice (n) of the tooth (*j*) can be derived as:

(5) $$\emptyset_{n,j}\left(t\right)=\left\{\begin{array}{cc} \emptyset_{n,j,0}\left(t\right), & \emptyset_{n,j,0}\left(t\right)\geq 0\\ \emptyset_{n,j,0}\left(t\right)+2\pi , & \emptyset_{n,j,0}\left(t\right)<0 \end{array}\right.,$$

where

(6) $$\emptyset_{n,j,0}\left(t\right)=\emptyset_{1,1}\left(t\right)-\left(j-1\right)\frac{2\pi r}{N}-\left(n-1\right)\frac{da\cdot \tan\left(\gamma \right)}{r}.$$

Substituting the value of $\emptyset_{n,j}\left(t\right)$ into Equation (1), the specific chip thickness of each cutting-edge slice is then obtained, and hence, the machining force acting on each part of the cutting edge can be estimated utilising Equation (3). Therefore, the resultant milling forces ($F_{x}(t)$ and $F_{y}(t)$) are:

(7) $$\left[\begin{array}{c} F_{x}\left(t\right)\\ F_{y}\left(t\right) \end{array}\right]=\sum_{n=1}^{S}\sum_{j=1}^{N}g\left(\emptyset_{n,j}\left(t\right)\right)\left[\begin{array}{cc} -cos\emptyset_{n,j}\left(t\right) & -sin\emptyset_{n,j}\left(t\right)\\ sin\emptyset_{n,j}\left(t\right) & -cos\emptyset_{n,j}\left(t\right) \end{array}\right]\left[\begin{array}{c} K_{tc}h_{n,j}\left(t\right)da+K_{te}da\\ K_{rc}h_{n,j}\left(t\right)da+K_{re}da \end{array}\right],$$

where S is the number of discretised slices of the cutter, N is the number of cutting teeth, and $g(\emptyset_{n,j}\left(t\right))$ represents the condition if the current slice (n) of the tooth (*j*) is engaged in cutting, which can be defined as:

(8) $$\left\{\begin{array}{cc} g\left(\emptyset_{n,j}\left(t\right)\right)=1, & \emptyset_{st}<\emptyset_{n,j}\left(t\right)<\emptyset_{ex}\\ g\left(\emptyset_{n,j}\left(t\right)\right)=0, & others \end{array}\right.,$$

where $\emptyset_{st}$ and $\emptyset_{ex}$ are the entry and exit angles of the tooth (*j*), respectively, and can be calculated based on the radial depth of cut (b), as follows:

(9) $$\left\{\begin{array}{cc} \emptyset_{st}\;=\;0\;and\;\emptyset_{ex}\;=\;\arccos\left(\frac{r-b}{r}\right), & up\;milling\\ \emptyset_{st}\;=\;\arccos\left(\frac{b-r}{r}\right)\;and\;\emptyset_{ex}\;=\;\pi , & down\;milling \end{array}\right..$$

In Equation (9), the radial depth of cut is a constant value, resulting in fixed values for the $\emptyset_{st}$ and $\emptyset_{ex}$ during the normal cutting process. However, as discussed in Section 1, the cutting engagement varies frequently when the cutter enters and exits the workpiece boundaries. Consequently, the resultant forces cannot be estimated utilising the above dynamics model. A novel approach that considers the variation in the cutting-engaged area is developed in the following section to provide an accurate prediction of the milling forces during these engagement and disengagement phases.

### 2.2. Process Analyses of Milling Tool Cutting into and out of Workpiece

Based on the variation in the chip thickness, the entire milling process can be classified into five steps:

Step 1. A rotating cutter moves to a workpiece in a particular feed direction. Along this direction, the cutter will not touch or cut the workpiece before a specific time interval. Thus, the chip thickness in this step is always zero, since no cutting is performed.

Step 2. A tooth of the cutter first touches the side of the workpiece and begins cutting the material. The cutting then starts, and the radial engagement is increased from a small area to the desired depth of cut. The challenges of calculating the chip thickness in this step come from two aspects: (i) the relative position between the workpiece boundary and the cutting teeth affects the chip and needs to be considered in the dynamics model; (ii) an accurate estimation of the varied radial engagement is required to calculate the chip thickness and predict the system dynamics.

Step 3. After the rotating cutter moves a sufficient distance into the workpiece and the radial immersion reaches the desired depth of cut, normal cutting starts and continues for a period of time until the cutter touches the other side of the workpiece. Therefore, during this step, the $\emptyset_{st}$ and the $\emptyset_{ex}$ defined in Equation (9) can be utilised to describe the cutting-engaged area, and the model proposed in Section 2.1 can be applied to predict the system dynamics.

Step 4. After normal cutting in the interior of the workpiece for a certain period of time, a tooth of the cutter first contacts the other side of the workpiece and cuts out of the material. The cutter then starts to exit the workpiece because there is insufficient material remaining in the current feed direction. Therefore, the variation in the chip thickness in this step arises from three aspects: (i) the dynamic displacements of the cutter relative to the workpiece caused by system vibrations; (ii) the non-neglected influence of the workpiece boundary on the formation of the chip; (iii) the frequent variations in the radial engagement and the remaining material.

Step 5. When the remaining material in the feed direction is completely removed by the cutter, the cutter moves away from the workpiece, and no cutting will occur. The chip will then not appear, and its thickness should be set to zero in the dynamics model.

The calculations of the chip thickness in Steps 2 and 4 are distinctly lacking; therefore, these calculations become the key factors in modelling the dynamics when the cutter enters and exits the sides of a workpiece.

**Figure 3:**
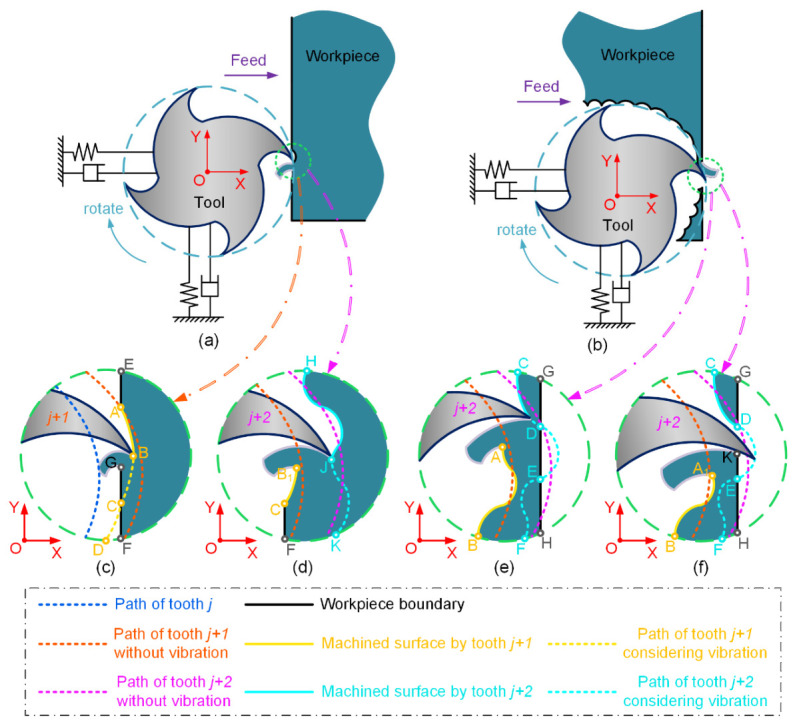
Schematic diagrams of the engagement and disengagement processes in milling operations, including: (**a**) the cutter entering the workpiece; (**b**) the cutter exiting the workpiece; chip formations of the cutter entering the workpiece boundary process, where (**c**) the side of the workpiece and (**d**) the surface machined by the previous tooth are removed; chip thickness calculations of the cutter cutting out the workpiece process, where (**e**) the previous tooth path and (**f**) the workpiece boundary need to be considered.

It should be noted that in Step 2, when a tooth of the cutter first touches and cuts the workpiece, the rotation angle of this tooth at this moment may not be the entry angle ($\emptyset_{st}$) defined for the normal cutting process. An example of this phenomenon is presented in Figure 3a. An up-milling operation with a four-tooth cutter at the beginning of the process is demonstrated, illustrating that the tooth first cuts into the workpiece at a rotation angle near the π/2. However, as defined in Equation (9), the $\emptyset_{st}$ of this up-milling process during normal cutting is 0; hence, it cannot be utilised to estimate the cutting engagement of this initial engagement process.

Similarly, in Step 4, when a cutting tooth first contacts the other side of the workpiece and starts to exit the material, the rotation angle of this tooth at this instant may not be the exit angle ($\emptyset_{ex}$) defined for the normal cutting. An example of this phenomenon is presented in Figure 3b, demonstrating that the tooth first cuts out of the workpiece at a rotation angle near the π/2 rather than the exit angle of the normal cutting. Therefore, to estimate the dynamics of the engagement and disengagement phases claimed in Steps 2 and 4, the relevant cutting engagement variations should be understood and modelled priorly.

In Step 2, the characteristics of chip formation can be divided into two main categories:

1. When a tooth of the cutter first cuts the workpiece, the movement of the previous tooth does not affect the chip thickness; however, the relative position between the workpiece boundary and the current tooth influences the chip formation and, hence, the chip becomes the material between the side of the workpiece and the path of the current tooth (*j*). An example of this case is presented in Figure 3c, in which the previous tooth (*j*) does not contact the workpiece (see its path represented by the blue dotted line), and the current tooth (*j + 1*) first cuts the material and creates the chip. The real path of the tooth (*j + 1*) is described by $\overset{⏜}{ABCD}$, indicating that the cutting edge removes the material at $\overset{⏜}{ABC}$ and cuts out from the workpiece at $\overset{⏜}{CD}$. Therefore, the chip is not the material between the paths of the previous tooth (*j*) and the current tooth (*j + 1*). Instead, the part of the workpiece boundary that is supposed to be cut by the current tooth (*j + 1*) (for instance, line GC in Figure 3c) needs to be considered in the calculation of the chip thickness for this process.
2. After the first tooth contacts the workpiece, the subsequent tooth both removes the machined surface produced by the previous tooth and cuts the remaining side of the workpiece. Therefore, the chip formation is affected by both the path of the previous tooth and the workpiece boundary. An example of such a case is demonstrated in Figure 3d, in which the previous tooth (*j + 1*) first cuts the material and leaves a surface; then, for the current tooth (*j + 2*), its real path can be represented by $\overset{⏜}{HJK}$, illustrating that both the machined surface left by the previous tooth (*j + 1*) (see $\overset{⏜}{B_{1}C}$ in Figure 3d) and the side of the workpiece (see line CF in Figure 3d) are supposed to be removed by the current tooth (*j + 2*). Consequently, when $\overset{⏜}{B_{1}C}$ is engaged in cutting, the produced chip is the material between the paths of the previous tooth (*j + 1*) and current tooth (*j + 2*); when the line CF is cut by the tooth *j + 2*, the chip becomes the material between the side of the workpiece (i.e., line CF) and the path of the current tooth.

To analyse the chip formation in Step 4, an example of a milling tooth contacting and exiting the side of the workpiece is presented in Figure 3e,f, and the movement of the tooth (*j + 2*) is described as the path $\overset{⏜}{CDEF}$, which can be divided into three parts:

1. When the current tooth is about to contact and exit the side of the workpiece, it typically begins cutting from the interior of the workpiece; before the cutting edge exits the material, the chip thickness generated by this tooth is the distance between the paths of the previous and current teeth. Therefore, when the tooth (*j + 2*) rotates to $\overset{⏜}{CD}$ (see Figure 3e), the relevant chip thickness can be calculated by Equation (1).
2. When the tooth starts to exit the side of the workpiece, since there is no sufficient material remaining in the cutting area, the chip thickness is defined as the distance between the workpiece boundary and the path of the previous tooth at this stage. Thus, when the tooth (*j + 2*) moves to $\overset{⏜}{DE}$ (see Figure 3f), a part of the workpiece boundary, line KE, should be utilised to estimate the chip thickness. It should be noted that after line KE is removed by tooth (*j + 2*), there is no material in this area, and hence, when the following tooth rotates to such a position, the corresponding chip thicknesses are always zero.
3. Under some milling conditions where large radial immersions occur, after a tooth exits the workpiece for a short time, its rotational motion may bring the tooth back to the workpiece and cut the material again. An example of this case is demonstrated in Figure 3f, where the tooth (*j + 2*) moves to $\overset{⏜}{DE}$, exits the workpiece temporarily, and then rotates to $\overset{⏜}{EF}$ and removes the material again, indicating that the relevant chip thickness is the distance between the paths of the previous tooth (*j + 1*) (i.e., $\overset{⏜}{AB}$ in Figure 3f) and current tooth (*j + 2*) (i.e., $\overset{⏜}{EF}$ in Figure 3f). It should be noted that this case only appears in machining conditions with large radial engagements and may not happen in milling processes with small radial depths of cuts.

Up to now, the continuously varying cutting engagement area has been fully defined and calculated. To further estimate the relevant chip thickness and simulate the system dynamics during these engagement and disengagement processes, the relative positions between the workpiece boundaries and cutting teeth need to be accurately predicted.

## 3. Dynamics Modelling of Milling Cutter Entering and Exiting Workpiece Boundaries

### 3.1. Cutting Engagement Calculation and Machining Dynamics Estimation When Milling Tool Cuts into Side of Workpiece

**Figure 4:**
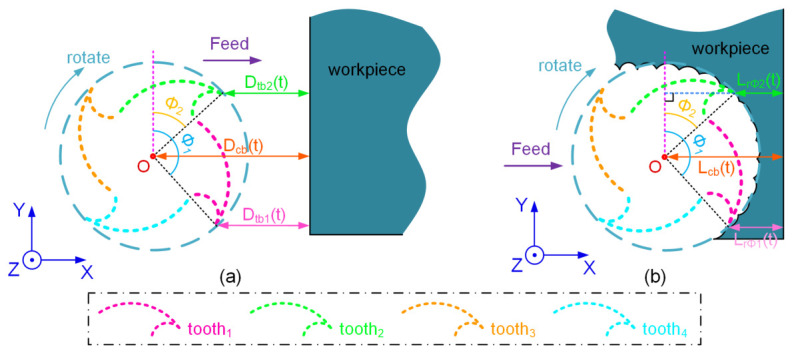
The relative positions of the workpiece boundaries and the cutting teeth when a cutter (**a**) enters (into) and (**b**) exits (from) the sides of the workpiece.

The position of the cutter relative to the workpiece before the cutter starts cutting is described in Figure 4a, where $\emptyset_{1}$ and $\emptyset_{2}$ are the rotation angles of tooth 1 and tooth 2, respectively. To detect the movements and cutting conditions of the tooth, the distances between the cutting edges and the side of the workpiece need to be calculated, as follows:

(10) $$\left\{\begin{array}{c} D_{tb1}(t)=D_{cb}(t)-rsin\emptyset_{1}\\ D_{tb2}(t)=D_{cb}(t)-rsin\emptyset_{2} \end{array},\right.$$

where $D_{tb1}(t)$ and $D_{tb2}(t)$ are the distances from teeth 1 and 2 to the left side of the workpiece at time t, respectively. $D_{cb}(t)$ denotes the distance between the tool centre and the workpiece’s left boundary and can be estimated by:

(11) $$D_{cb}\left(t\right)=\left\{\begin{array}{c} \;\;\;\;\;\;\;\;\;\;\;\;\;\;\;\;D_{0}-ft,\;\;before\;the\;cutter\;touches\;the\;workpiece\\ D_{0}-ft-\sum_{i=t_{1}}^{t}x_{i},\;\;after\;the\;cutter\;first\;cuts\;the\;material \end{array}\right.,$$

where $D_{0}$ is the distance from the initial position of the tool centre to the left side of the workpiece; f is the feed speed (mm/s); t is a certain time value after the cutter moves; $t_{1}$ is the time that the tool first cuts the workpiece; and $x_{i}$ is the dynamic displacement of the cutter relative to the workpiece along the feed direction.

Based on the calculations of the relative distances between the cutting edges and the workpiece boundary utilising Equation (10), a solution can be derived to estimate the cutting conditions of the tooth when the milling cutter enters the side of the workpiece:

- If the distance from the tooth to the workpiece boundary is positive, then the tooth is located outside the workpiece, and no cutting is performed consequently. The chip thickness and the forces corresponding to this tooth are zero.
- If the distance from the current tooth to the side of the workpiece is negative, then the tooth has crossed the workpiece boundary and removed a certain amount of material, resulting in a series of chip thickness values, which can be calculated according to the following:

1. If the side of the workpiece is involved in the chip formation of a tooth at a particular instant, the chip thickness should be the distance from the workpiece boundary to the path of that tooth. Therefore, the chip thickness can be estimated as:

(12) $$h_{j}\left(t\right)=\frac{ft+\sum_{i=t_{0}}^{t}x_{i}+rsin\emptyset_{j}\left(t\right)-D_{0}}{sin\emptyset_{j}\left(t\right)},$$

where $t_{0}$ is the time that the cutter starts to move to the workpiece, and the dynamic displacement ($x_{i}$) is defined as:

(13) $$x_{i}=\left\{\begin{array}{c} \;\;\;\;\;\;\;\;\;\;\;\;\;\;\;\;\;\;\;\;\;\;0,\;\;\;\;\;\;\;\;before\;the\;cutter\;touches\;the\;workpiece\\ x_{i-1}+{\dot{x}}_{i-1}dt,\;\;\;\;\;\;\;\;after\;the\;cutter\;first\;cuts\;the\;material \end{array}\right.,$$

where the velocity from the previous time step, ${\dot{x}}_{i-1}$, can be similarly calculated as:

(14) $${\dot{x}}_{i-1}=\left\{\begin{array}{c} \;\;\;\;\;\;\;\;\;\;\;\;\;\;\;\;\;\;\;\;\;\;0,\;\;\;\;\;\;\;\;before\;the\;cutter\;touches\;the\;workpiece\\ {\dot{x}}_{i-2}+{\ddot{x}}_{i-2}dt,\;\;\;\;\;\;\;\;after\;the\;cutter\;first\;cuts\;the\;material \end{array}\right.,$$

where the acceleration (${\ddot{x}}_{i-2}$) can be derived from Equation (2):

(15) $${\ddot{x}}_{i-2}=\left\{\begin{array}{c} \;\;\;\;\;\;\;\;\;\;\;\;\;\;\;\;\;\;\;\;\;\;\;\;\;\;\;\;\;\;\;\;\;\;\;0,\;\;\;\;\;\;\;\;before\;the\;cutter\;touches\;the\;workpiece\\ \frac{F_{x}-c_{x}{\dot{x}}_{i-3}+k_{x}x_{i-3}}{m_{x}},\;\;\;\;\;\;\;\;after\;the\;cutter\;first\;cuts\;the\;material \end{array},\right.$$

where $c_{x}$ and $m_{x}$ are the damping and mass of the system in the x direction, respectively.

**Figure 5:**
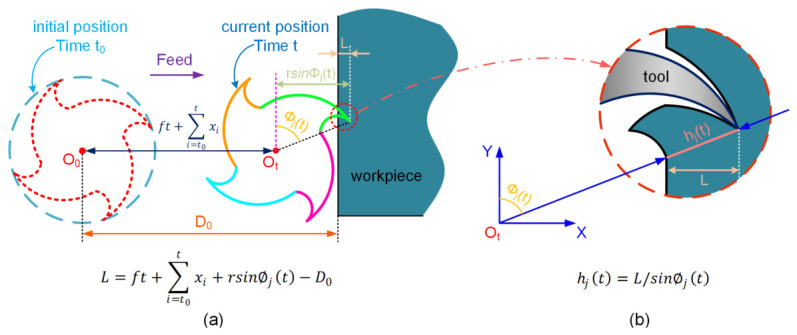
Schematic diagrams of estimating the chip thickness when a milling tool enters (into) the workpiece boundary, including a (**a**) general view and (**b**) partial view.

Schematic diagrams of this chip thickness estimation strategy are demonstrated in Figure 5, indicating that the distance between the centres of the cutter at its initial position and current position, i.e., $O_{0}O_{t}$, is $ft+\sum_{i=t_{0}}^{t}x_{i}$ (see Figure 5a). Therefore, the length that the tooth cuts into the material along the feed direction can be calculated utilising the formula presented in Figure 5a, and hence, the chip thickness ($h_{j}\left(t\right)$) can be estimated through the formula provided in Figure 5b, leading to the final equation for predicting the $h_{j}\left(t\right)$ shown in Equation (12).

An example of calculating the chip thickness in this case is presented in Figure 3c, where a part of the workpiece boundary (i.e., line GC) is engaged in cutting, and hence, Equation (12) is needed to estimate the relevant chip thickness for this condition.

2. If the side of the workpiece corresponding to the current cutting area has been removed by the previous tooth, then a wave surface is formed, and thus the chip becomes the material between the wave surfaces left by the previous and current teeth. The chip thickness can then be calculated by Equation (1).

For instance, in Figure 3d, a part of the workpiece boundary is cut by the previous tooth (*j + 1*), and a wave surface ($\overset{⏜}{B_{1}C}$) is left. When the current tooth (*j + 2*) rotates to the same rotation angles, the side of the workpiece has already been removed, and only the $\overset{⏜}{B_{1}C}$ is about to be cut. Equation (1) can thus be utilised to predict the chip thickness in this case.

It should be noted that in Equation (12), the workpiece boundary is assumed to be a straight line perpendicular to the feed direction. For other non-rectangular boundaries, a differential approach can be used to approximate them with rectangular infinitesimal elements.

### 3.2. Dynamics Modelling of Milling Cutter Exiting Workpiece Boundary

**Figure 6:**
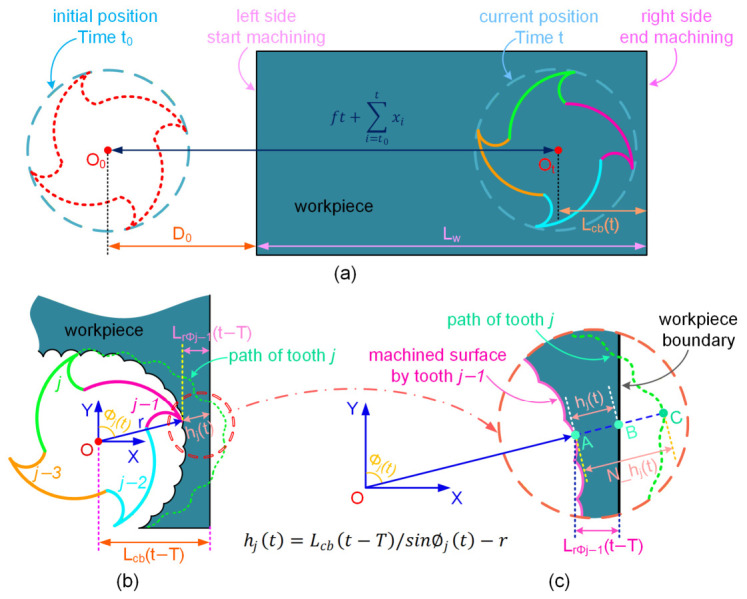
Schematic diagrams of estimating the chip thickness when a milling tool exits (from) the workpiece boundary, including (**a**) the relative distances between the cutter’s positions and the sides of the workpiece, and a (**b**) general view and (**c**) partial view of the chip formation when the tooth (*j*) cuts out the side of the workpiece.

The relative position between the tool and the workpiece when a cutter is about to exit the side of the workpiece is demonstrated in Figure 4b. Similarly, to estimate the instants when the cutting edges exit the workpiece boundary and model the relevant machining dynamics, the lengths of the remaining material along the feed direction corresponding to the rotation angles $\emptyset_{1}$ and $\emptyset_{2}$, i.e., $L_{r\emptyset 1}(t)$ and $L_{r\emptyset 2}(t)$, are defined as follows, respectively:

(16) $$\left\{\begin{array}{c} L_{r\emptyset 1}(t)=L_{cb}(t)-rsin\emptyset_{1}\\ L_{r\emptyset 2}(t)=L_{cb}(t)-rsin\emptyset_{2} \end{array}\right.,$$

where $L_{cb}(t)$ is the distance between the tool centre and the right side of the workpiece, as illustrated in Figure 6a, and can be calculated as:

(17) $$L_{cb}\left(t\right)=\left(D_{0}+L_{w}\right)-\left(ft+\sum_{i=t_{0}}^{t}x_{i}\right),$$

where $L_{w}$ is the length of the workpiece; $\left(D_{0}+L_{w}\right)$ represents the distance from the centre of the cutter at its initial position to the farther boundary of the workpiece; $\left(ft+\sum_{i=t_{0}}^{t}x_{i}\right)$ is the distance that the cutter has moved; and $L_{cb}(t)$ becomes the length from the tool centre at its current position to the side of the workpiece (see Figure 6a).

According to these definitions, when the tooth (*j*) is about to exit the workpiece boundary at the rotation angle $\emptyset_{j}\left(t\right)$, the relevant chip thickness ($h_{j}\left(t\right)$) should be the length between the machined surface by the previous tooth (*j* − *1*) and the side of the workpiece (see Figure 6b). To estimate the chip thickness ($h_{j}\left(t\right)$) of the tooth (*j*), it is necessary to first calculate the length of the remaining material along the feed direction when the previous tooth (*j* − *1*) rotates to the rotation angle $\emptyset_{j-1}\left(t-T\right)$ (which is equal to the rotation angle $\emptyset_{j}\left(t\right)$), as follows:

(18) $$L_{r\emptyset_{j-1}}\left(t-T\right)=L_{cb}\left(t-T\right)-rsin\emptyset_{j}\left(t\right).$$

Since the chip thickness corresponding to the tooth (*j*) at the rotation angle $\emptyset_{j}\left(t\right)$ is the length of the remaining material along the radial direction (see Figure 6c), $h_{j}\left(t\right)$ can be estimated as:

(19) $$h_{j}\left(t\right)=\frac{L_{r\emptyset_{j}-1}\left(t-T\right)}{sin\emptyset_{j}\left(t\right)}.$$

Substituting Equation (18) into Equation (19), the relevant chip thickness corresponding to the rotation angle $\emptyset_{j}\left(t\right)$ during the process of the tooth (*j*) exiting the workpiece boundary can be calculated as follows.

(20) $$h_{j}\left(t\right)=\frac{L_{cb}\left(t-T\right)}{sin\emptyset_{j}\left(t\right)}-r.$$

It should be noted that in Figure 6c, $N\_h_{j}\left(t\right)$ represents the nominal chip thickness predicted by the existing model for the normal cutting process, and it is defined as the distance between adjacent tooth paths and can be calculated by Equation (1). However, when tooth j exits the workpiece boundary, since insufficient material remains in the cutting area, $N\_h_{j}\left(t\right)$ exceeds the length of the remaining workpiece along the radial direction (see Figure 6c). Therefore, the actual chip thickness ($h_{j}\left(t\right)$) is smaller than the $N\_h_{j}\left(t\right)$ and should be estimated by Equation (20). Also, since the $h_{j}\left(t\right)$ calculated by Equation (20) represents the length of the remaining material in the radial direction corresponding to the rotation angle $\emptyset_{j}\left(t\right)$, if it is larger than the nominal chip thickness ($N\_h_{j}\left(t\right)$), this indicates that the material in the cutting area is sufficient and, hence, the current cutting condition is normal cutting. The chip thickness should then be calculated by Equation (1).

Based on the above analysis of the chip thickness estimation, the dynamics of a milling system when the cutter exits the side of the workpiece can be predicted as follows:

1. The distance between the current and previous tooth paths along the radial direction are calculated at the rotation angle $\emptyset_{j}\left(t\right)$ utilising Equation (1).
2. The length of the remaining workpiece along the radial direction is calculated through Equation (20). It should be noted that Equation (17) must be applied to obtain the $L_{cb}\left(t-T\right)$ in Equation (20).
3. If the distance calculated in Step 1 exceeds the length obtained from Step 2, the current tooth cuts out from the side of the workpiece and, hence, the actual chip thickness should be predicted using Equation (20). Otherwise, the current tooth is still involved in the normal cutting, and thus the chip thickness needs to be calculated by Equation (1).
4. After a tooth exits the workpiece boundary at the rotation angle $\emptyset_{j}\left(t\right)$, $\emptyset_{j}\left(t\right)$ should be marked, since no material remains at this rotation angle. Then, when the following teeth rotate to the same angle, the relevant chip thicknesses are always zero.
5. Based on the calculated chip thickness, the cutting forces can be predicted by Equation (7), and then the system dynamics can be estimated by Equations (2) and (13).

The above procedure is repeated until the whole milling cutter exits the workpiece, and thus the cutting process is finished.

Because the material is removed from the workpiece by a solid cutter for the milling process with some mechanical principles, the shape of the formed object is geometrically dependent on the movement of the tool [30,31]. Therefore, by estimating the trajectories of the cutting teeth, the profiles of the machined surface can be predicted.

The trajectories of the cutting teeth are ultimately determined by both the tool feed motion and tool vibration. Thus, by calculating the tool’s feed and predicting the cutter vibration, the tool path can be determined, which enables the accurate prediction of the machined surface profile.

**Figure 7:**
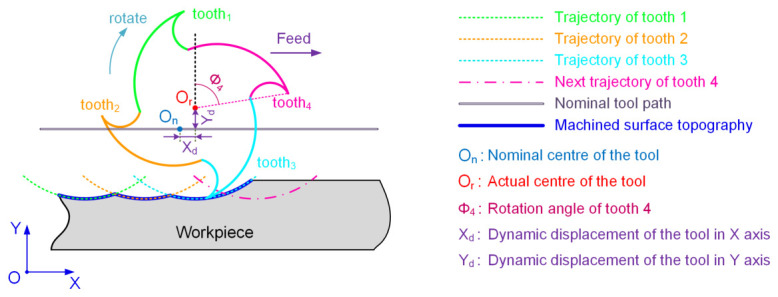
Schematic diagrams of estimating machined surface topography based on calculated tool teeth trajectories.

As shown in Figure 7, the profiles of the machined surface topography can be predicted based on the calculated tool teeth trajectories:

1. At each moment, the real-time nominal position of the tool centre (i.e., $O_{n}$) is calculated based on the feed speed.
2. Based on Step 1, the actual centre of the tool (i.e., $O_{r}$) is determined by combining the nominal centre of the tool (i.e., $O_{n}$) with the dynamic displacement (i.e., $X_{d}$ and $Y_{d}$) caused by the cutting vibration.
3. Based on the actual centre of the tool (i.e., $O_{r}$), and considering the rotation angle of each tooth (for example, $\emptyset_{4}$ in Figure 7), the trajectories of each cutting tooth can be calculated.
4. Then, the intersection of each cutting tooth’s trajectory with the workpiece generates the final machined surface topography.

## 4. Experimental Validation of Developed Approach on Estimating Dynamics of Milling Tool Entering and Exiting Sides of Workpiece

In the following, the analyses of the experimental and simulated milling forces are first presented to verify the developed dynamics model, with the relevant machined surfaces also provided to demonstrate the vibration marks, and then the verification of the developed approach regarding machined surface estimation is reported. Based on the accurate simulation of the milling dynamics by this presented model, a strategy that enables vibration suppression by selecting the proper machining parameters is reported. Both the cutting forces and machined surfaces of the selected milling procedure are provided to illustrate that the developed strategy has the ability to avoid vibrations happening in the whole milling process.

### 4.1. Experimental Setup

The relevant milling trials were conducted using an aluminium alloy workpiece, together with a four-tooth ∅10 mm helical end milling cutter (helix angle: $38^{{}^{\circ}}$) and a four-axis milling machine (YHVT850Z machining centre, Qinchuan Machine Tool Group, Baoji, China). A miniature accelerometer (3225F1T, Dytran, Chatsworth, CA, USA) and a hammer (9722A500, Kistler, Winterthur, Switzerland) were applied to perform the hammer test to obtain the modal parameters of the milling system. A three-component dynamometer (9257B, Kistler, Winterthur, Switzerland) and an optical 3D surface measurement system (Alicona IFM-G5, Alicona, Graz, Austria) were utilised to capture the cutting forces and measure the machined surfaces, respectively. During the experiments, milling forces acting on the workpiece in the x ($F_{x}$) and y ($F_{y}$) directions were measured, and the feed direction was parallel to the $F_{x}$.

### 4.2. Validation of Developed Approach During Cutter–Workpiece Engagement and Disengagement

**Figure 8:**
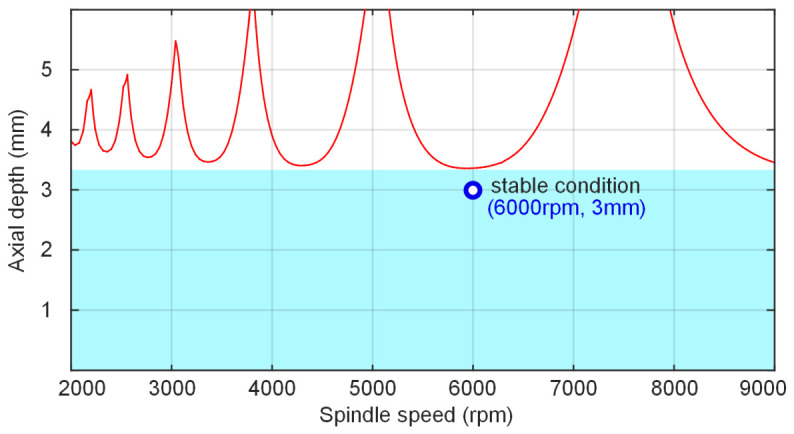
Stability lobes of proposed milling system for slotting operation.

To obtain the system modal parameters and calibrate the cutting force coefficients, hammer tests and slot milling trials at different feed speeds were conducted first. The extracted modal parameters are listed in Table 1, and the calibrated force coefficients were: $K_{tc}=297.68\;MPa$; $K_{rc}=158.74\;MPa$; $K_{te}=1.2840\times 10^{4}\;N/m$; $K_{re}=1.0103\times 10^{4}\;N/m$. Then, the stability lobes of this milling system for the slotting procedure were calculated and visualised (see Figure 8).

**Figure 9:**
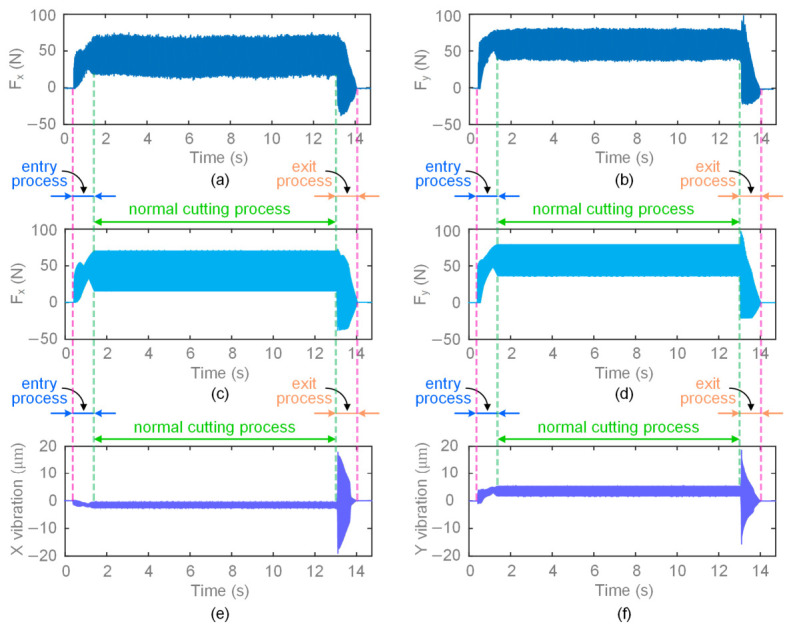
Verification of developed approach with regard to cutting dynamics prediction, including: (**a**) experimental forces on *x* axis; (**b**) experimental forces on *y* axis; (**c**) simulated forces on *x* axis; (**d**) simulated forces on *y* axis; (**e**) simulated dynamic displacement of tool on *x* axis; (**f**) simulated dynamic displacement of tool on y axis.

A stable cutting condition (spindle speed: 6000 rpm; axial depth of cut: 3 mm) was arbitrarily selected from the stable area to carry out a milling trial under the feed speed of 300 mm/min. The experimental milling forces in the *x (*$F_{x}$*)* and *y (*$F_{y}$*)* directions are presented in Figure 9a,b, respectively. The simulated milling forces in the *x (*$F_{x}$*)* and *y (*$F_{y}$*)* directions are separately presented in Figure 9c,d. Additionally, the system vibrations in the x and y directions, which can be reflected in the dynamic displacements between the tool and workpiece, are demonstrated in Figure 9e,f, respectively. These results substantiate the following conclusions:

1. The entire milling process can be divided into three distinct stages, i.e., the entry process, normal cutting process, and exit process. Each stage has distinct characteristics, and the differences between the adjacent stages are sufficiently clear. The simulation results of the cutting forces exhibited excellent agreement with the experimental data at these three stages.
2. The experimental forces (Figure 9a,b) demonstrate that the normal cutting process was stable, with the simulated forces (Figure 9c,d) also reflecting this conclusion, and the system’s vibrations (Figure 9e,f) were maintained within a relatively small range.
3. A stable cutting process could also be found in the engagement process when the cutter entered the side of the workpiece, which can be observed from the entry process in the forces presented in Figure 9; the system’s vibrations also validate this finding.
4. During the cutter exiting the workpiece boundary process, the system experienced a significant oscillation, with the cutting force exceeding the previous peak and the amplitude increasing abruptly (see Figure 9a,b). Consequently, system vibrations also abruptly intensified (see Figure 9e,f).

**Figure 10:**
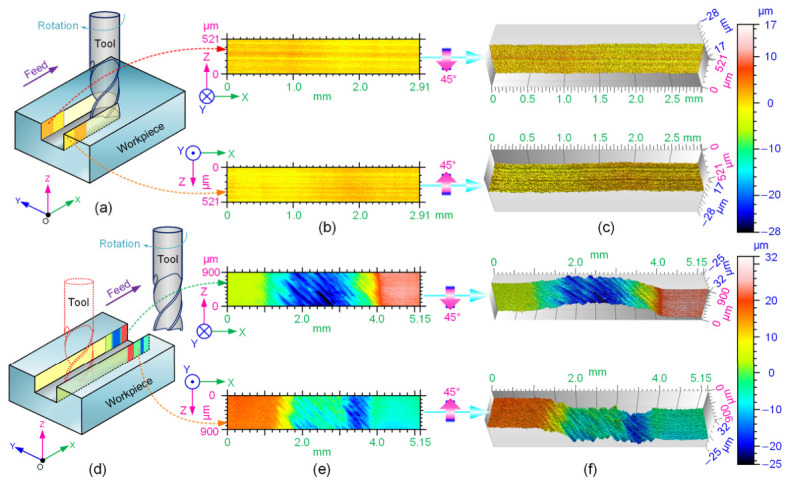
The surfaces formed by a slot milling procedure, including: (**a**) a schematic diagram of the cutter entering the side of the workpiece process; (**b**) 2D and (**c**) 3D measurement results of the surface formed by the cutter entering the side of the workpiece process; (**d**) a schematic diagram of the cutter exiting the workpiece boundary process; (**e**) 2D and (**f**) 3D measurement results of the surface produced by the cutter exiting the workpiece boundary process.

To further validate the above finding, the measurement results of the machined surface are illustrated in Figure 10. It can be observed that the machined surface exhibits a relatively smooth profile (Figure 10b,c) for the cutter entering the side of the workpiece process (Figure 10a), indicating a stable machining process. For the cutter exiting the workpiece boundary process (Figure 10d), visible machining marks can be observed (Figure 10e,f), reflecting significant vibrations experienced by the system during this process. These findings align with the conclusions presented in Figure 9.

Following the accurate prediction of cutting forces and vibrations utilising the proposed model, it is then possible to further predict the tool’s motion trajectory based on the calculated cutting dynamics. Consequently, the machined surface topography can be estimated.

**Figure 11:**
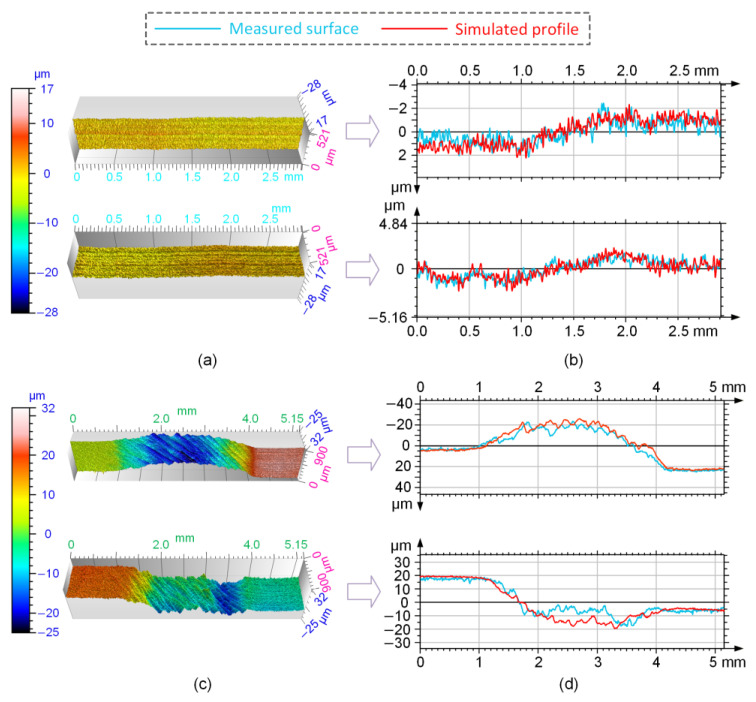
Verification of developed approach regarding machined surface estimation, including: (**a**) 3D measurement results of surface; (**b**) 2D profiles of measured and simulated surfaces produced by cutter entering side of workpiece process; (**c**) 3D measurement results of surface; and (**d**) 2D profiles of measured and simulated surfaces produced by cutter exiting workpiece boundary process.

The measured three-dimensional surfaces in the cutter entering the side of the workpiece process are presented in Figure 11a, and the two-dimensional contour projections obtained from three-dimensional measurements are shown in Figure 11b. Furthermore, the three-dimensional measurements of the machined surface when the cutter exited the workpiece boundary process are presented in Figure 11c, and the two-dimensional contour projections obtained from three-dimensional measurements are shown in Figure 11d. As a comparison, the simulated surface topographies are also demonstrated in Figure 11b,d. It could be observed that significant vibration marks were generated during the process of the cutter exiting the workpiece boundary, and the simulation results accurately reflect this occurrence. Furthermore, the simulations demonstrate good agreement with the experimental measurements, thereby validating the effectiveness of the proposed model.

### 4.3. Milling Parameter Optimisation Based on Developed Approach

As illustrated in the preceding section, even when selecting stable milling parameters from the stability lobe, stability cannot be guaranteed throughout the entire machining process. Therefore, by leveraging the developed approach’s ability to predict the machining dynamics of the entire milling process, a method for optimising milling parameters can be established.

**Figure 12:**
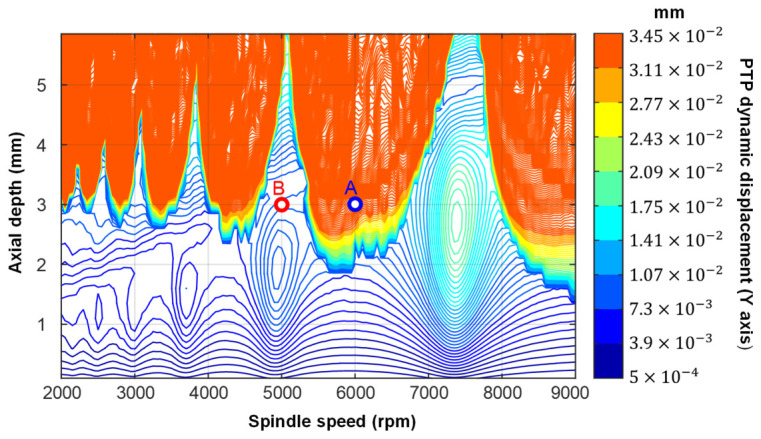
Two-dimensional contour of peak-to-peak dynamic displacement between cutter and workpiece.

Since the developed approach could predict cutting forces and dynamic displacements between the tool and workpiece throughout the entire milling process, the stability could be determined by plotting the peak-to-peak (PTP) dynamic displacement values for a range of spindle speeds and axial-depth-of-cut combinations. Therefore, by plotting a PTP dynamic displacement diagram within a specific range of spindle speeds and cutting depths, the stability of the machining conditions could be assessed, and appropriate milling parameters could be selected subsequently. As shown in Figure 12, the milling condition selected in the preceding section (spindle speed: 6000 rpm; axial depth of cut: 3 mm), which is marked as point A, is in a region of high PTP dynamic displacement values, indicating that this machining parameter would result in significant machining vibrations. This finding matches the validation in the preceding section.

**Figure 13:**
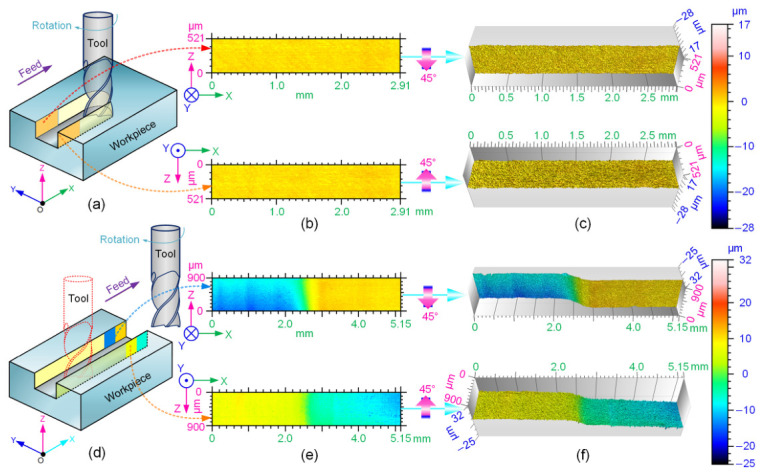
Surfaces generated by slotting operation under selected milling parameters, including: (**a**) schematic diagram of cutter entering side of workpiece process; (**b**) 2D and (**c**) 3D measurement results of surface formed by cutter entering side of workpiece process; (**d**) schematic diagram of cutter exiting workpiece boundary process; (**e**) 2D and (**f**) 3D measurement results of surface produced by cutter exiting workpiece boundary process.

Furthermore, to optimise the milling stability in the entire machining process, a stable milling condition can be selected from Figure 12 as an example. The cutting condition with a spindle speed of 5000 rpm and an axial depth of cut of 3 mm, marked as point B, lies within a region of low PTP dynamic displacement values, indicating that this machining parameter would result in a stable milling process. The machined surfaces generated by this milling condition are presented in Figure 13. Compared to Figure 10, the optimised parameters achieved a superior machined surface finish whilst significantly suppressing machining vibrations, and the machined surface demonstrated no pronounced vibration marks. Therefore, the effectiveness of the optimised machining parameters has been verified.

### 4.4. Further Verification of Developed Approach in Partial-Immersion Milling Test

**Figure 14:**
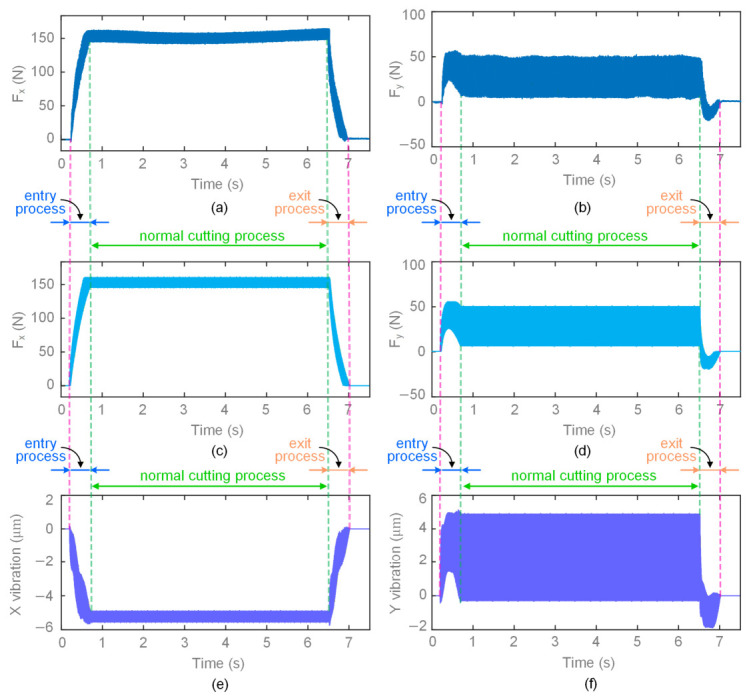
Validation of developed approach in half-immersion up-milling test, including: (**a**) experimental forces on x axis; (**b**) experimental forces on y axis; (**c**) simulated forces on x axis; (**d**) simulated forces on y axis; (**e**) simulated dynamic displacement of tool on x axis; (**f**) simulated dynamic displacement of tool on y axis.

To further validate the developed approach, an up-milling condition with a spindle speed of 6000 rpm, a feed speed of 600 mm/min, an axial depth of cut of 8 mm, and a radial depth of cut of 5 mm (i.e., half-immersion up milling) was selected to conduct the verification. The experimental milling forces in the *x (*$F_{x}$*)* and *y (*$F_{y}$*)* directions are presented in Figure 14a,b, respectively. The simulated milling forces in the *x (*$F_{x}$*)* and *y (*$F_{y}$*)* directions are separately presented in Figure 14c,d. Additionally, the system vibrations in the x and y directions, which can be reflected in the dynamic displacements between the tool and workpiece, are demonstrated in Figure 14e,f, respectively.

It can be observed from Figure 14 that the predicted cutting forces are in good agreement with the experimental values, and the cutting system experienced significant oscillations during cutter–workpiece engagement and disengagement. In particular, in the Y-direction, the cutting force decreases rapidly during the process of the cutter exiting the workpiece boundary, and the $F_{y}$ may even become negative, causing the dynamic displacement between the tool and workpiece to reverse, thereby affecting the consistency of the whole machining process.

## 5. Conclusions

A novel modelling framework to estimate the variability in the system dynamics when milling cutters enter and exit workpiece boundaries has been proposed in this paper. The mechanics of these engagement and disengagement processes have been understood and modelled by analysing the instantaneous cutting-engaged area at each instant of time, resulting in a methodology that is capable of accurately predicting cutting forces and machined surface profiles in these processes and, hence, providing a strategy for selecting appropriate milling parameters to avoid vibrations. This paper’s main contributions can be concluded as follows:

- The so-called ‘stable parameters’ obtained from the stability lobe only work for normal milling conditions and cannot guarantee stable machining processes when workpiece boundaries are engaged in cutting. It has been proven that during the entire milling process, even when a stable machining condition can be observed at normal cutting, vibrations may appear during the processes of the cutter entering (into) and exiting (from) the workpiece boundaries, limiting the processing performance and surface integrity.
- The frequent variation in the cutting-engaged area during the cutter–workpiece engagement and disengagement stages has been understood and modelled, leading to a novel dynamics model that is capable of accurately estimating milling forces and surface integrity. The validations demonstrate that the simulated and experimental forces have satisfactory agreements, and the predicted surface profiles are consistent with the measured data.
- A dedicated strategy that is able to evaluate the dynamic displacements of the cutter relative to the workpiece under various milling conditions is presented to suggest appropriate cutting parameters for vibration avoidance, providing optimised parameters that resulted in a stable machining process and step-improved surface quality during the experimental verification.

The findings proposed in this paper indicate that the developed approach could be utilised to predict cutting forces and surface profiles during the processes of the milling cutter entering and exiting the workpiece boundaries. Furthermore, the results of this study not only allow for an understanding of the system dynamics and the avoidance of vibrations but also open avenues for clarification of other scientific queries, such as energy consumption estimation, cutting parameter optimisation and tool wear prediction for the entire milling process.

## Figures and Tables

**Table 1:** Modal parameters of milling system.

Direction	$\omega_{n}\;(Hz)$	ξ (%)	k (N/m)
X	1054.5	2.774	$2.9140\times 10^{7}$
Y	976	4.022	$1.5896\times 10^{7}$

## Data Availability

The data presented in this study are available within the article.

## Nomenclature

$h_{j}\left(t\right)$	Chip thickness created by tooth *j* at time *t*	$\emptyset_{j}\left(t\right)$	Rotation angle of tooth *j* at time *t*
$f_{j}$	Feed speed of tooth *j*	T	Tooth period
$\upsilon_{j}\left(t\right)$	Dynamic displacement of current tooth	$\upsilon_{j}\left(t-T\right)$	Dynamic displacement of previous tooth
$\xi_{x}$	Damping ratio on *x*-axis	$\xi_{y}$	Damping ratio on *y*-axis
$\omega_{nx}$	Natural frequency on *x*-axis	$\omega_{ny}$	Natural frequency on *y*-axis
$k_{x}$	Spring stiffness on *x*-axis	$k_{y}$	Spring stiffness on *y*-axis
$F_{x}$	Force acting in x-direction	$F_{y}$	Force acting in y-direction
$F_{t,j}(t)$	Tangential force acting on tooth *j* at time *t*	$F_{r,j}(t)$	Radial force acting on tooth *j* at time *t*
$K_{tc}$	Cutting force coefficient in tangential direction	$K_{rc}$	Cutting force coefficient in radial direction
$K_{te}$	Edge constant in tangential direction	$K_{re}$	Edge constant in radial direction
a	Axial depth of cut	γ	Helix angle of cutter
$\emptyset_{n,j}\left(t\right)$	Rotation angle corresponding to slice *n* of tooth *j*	N	Number of tool flutes
r	Radius of cutter	$\emptyset_{st}$	Entry angle
$\emptyset_{ex}$	Exit angle	b	Radial depth of cut
$D_{tb}(t)$	Distance from tooth to left boundary of workpiece	$D_{cb}(t)$	Distance between cutter’s centre and workpiece’s left boundary
$D_{0}$	Distance from initial position of tool’s centre to left side of workpiece	x	Dynamic displacement of cutter relative to workpiece along feed direction
f	Feed speed (mm/s)	$t_{1}$	Time that cutter first cuts workpiece
$t_{0}$	Time that cutter starts to move to workpiece	$\dot{x}$	Dynamic velocity of milling system
$\ddot{x}$	Dynamic acceleration of milling system	$c_{x}$	Damping coefficient on *x*-axis
$m_{x}$	Mass on *x*-axis	$L_{r\emptyset }(t)$	Length of remaining material along feed direction
$L_{cb}(t)$	Distance between tool centre and right side of workpiece	$L_{w}$	Length of workpiece
